# Sleep Quality, Insomnia, Anxiety, Fatigue, Stress, Memory and Active Coping during the COVID-19 Pandemic

**DOI:** 10.3390/ijerph19094940

**Published:** 2022-04-19

**Authors:** Jaber S. Alqahtani, Saad M. AlRabeeah, Abdulelah M. Aldhahir, Rayan Siraj, Yousef S. Aldabayan, Saeed M. Alghamdi, Abdullah S. Alqahtani, Sulaiman S. Alsaif, Abdallah Y. Naser, Hassan Alwafi

**Affiliations:** 1 Department of Respiratory Care, Prince Sultan Military College of Health Sciences, Dammam 34313, Saudi Arabia; saad.alrabeeah@gmail.com (S.M.A.); rt0075@hotmail.com (A.S.A.); 2 Respiratory Therapy Department, Faculty of Applied Medical Sciences, Jazan University, Jazan 45142, Saudi Arabia; aldhahir.abdulelah@hotmail.com; 3 Department of Respiratory Care, King Faisal University, Al Ahsa 31982, Saudi Arabia; rayan_as18@hotmail.com (R.S.); rt.yousef1@hotmail.com (Y.S.A.); 4 National Heart and Lung Institute, Imperial College London, London SW7 2BX, UK; s.alsaif18@imperial.ac.uk; 5 Respiratory Care Program, Faculty of Applied Medical Sciences, Umm Al-Qura University, Makkah 21961, Saudi Arabia; 6 Rehabilitation Health Sciences Department, College of Applied Medical Sciences, King Saud University, Riyadh 11362, Saudi Arabia; 7 Department of Applied Pharmaceutical Sciences and Clinical Pharmacy, Faculty of Pharmacy, Isra University, Amman 11622, Jordan; abdallah.naser@iu.edu.jo; 8 Faculty of Medicine, Umm Al Qura University, Mecca 21514, Saudi Arabia; hhwafi@uqu.edu.sa

**Keywords:** sleep quality, insomnia, COVID, fatigue

## Abstract

Background: The current study aimed to evaluate the impact of the coronavirus (COVID-19) pandemic on sleep quality, insomnia, anxiety, stress, fatigue and active coping in the United States. Methods: This was a cross-sectional study using a publicly available database taken from the Boston College COVID-19 Sleep and Well-Being Dataset. We have selected the most recent data that included information about sleep quality and other measures, including insomnia, anxiety, stress, fatigue and coping, collected between 22 February–8 March 2021. Results: A total of 476 subjects were included in the analysis. The mean (SD) age of the study population was 38.8 (17.8) years, and there were more females (85%) than males. The population had a mean (SD) score of the Pittsburgh Sleep Quality Index (PSQI) of 6 (3.2), with 65% having the prevalence of poor sleep quality (defined as PSQ ≥ 5; *n* = 311). The mean (SD) score for Insomnia Severity Index (ISI) was 6.9 (5.2), with 55 subjects (11.5%) having clinical insomnia (defined as ISI ≥ 15); of whom 9% had severe clinical insomnia. There were positive correlations between PSQI and ISI (r = 0.76, *p* < 0.001), PROMIS fatigue scale (r = 0.53, *p* < 0.001), Generalized Anxiety Disorder-7 (GAD-7) (r = 0.46, *p* < 0.001), and Perceived Stress Scale (PSS) (r = 0.44, *p* < 0.001). The PSQI was inversely correlated with the John Henryism Active Coping Scale (JHACS) and memory scale. In the multivariate regression model, JHACS, ISI, fatigue, PSS and GAD-7 were significant predictors of PSQI, and these variables accounted for 62% of the variance of PSQI, adjusted for age and gender. Conclusion: An important contribution to the literature is made by this research, which demonstrates the significant prevalence of poor sleep quality and its association with insomnia and other mental and physical well-being. It also underlines the need to prioritise policy and public health efforts to address sleep issues that have substantial health and economic effects for both individuals and the population at large.

## 1. Introduction

Coronavirus disease 2019 (COVID-19) has been found to be capable of rapid transmission and progressive development of several aggressive variants, which can lead to increased severity and mortality globally [1]. Lythgoe and Middleton (2020) described the COVID-19 symptoms to be variable [2], including fever, cough, difficulty in breathing, loss of smell, loss of taste, fatigue and other flu-like symptoms. Statistical representations and real-world observations show that around one-third of infected individuals do not develop any perceivable difficulties or symptoms. However, 15% of those with symptoms develop severe and potentially fatal complications or conditions, with older people at higher risks [3]. Due to the global outbreak, massive infection rates and the high number of fatalities, lockdowns and restrictions continue to be implemented and recommended by global entities and authorities. 

While the physiological effects of the disease are prominently documented and distinguished, the psychological and mental effects are equally concerning and evident. Despite the fact that the disease itself does not directly target the public’s mental health, various psychosocial factors and conditions massively impact mental health and general wellbeing [4]. Mental health is often overlooked when discussing the segments affected by the ongoing global pandemic. While considerable progress has been made in the understanding and handling of mental health, especially during times of crisis, lapses in mental healthcare and services are still found [5]. According to Moreno et al. (2020) [6], uncertainty with the resolution and adaptation of the COVID-19 pandemic could potentially increase mental health risks. 

Lockdowns, restrictions, economic recessions, physical distancing, and other strategies are considered major factors in the potential incitation and exacerbation of mental health conditions and symptoms, especially anxiety. Furthermore, the threat of infection, along with the subsequent mental distress of symptoms and recovery and isolation due to the disease itself, are slated to further increase the intensity of anxiety and psychological instability. Many studies in the Middle East region have identified multiple detrimental social and psychological implications of the epidemic among all sectors of the public, including healthcare professionals, university students, and the general public [7,8,9,10].

Kola (2020) explained that further shifts in mental health services need to be observed [5]. The availability and adaptability of mental healthcare within the pandemic setting require adequate concern to adjust and compensate for the potential increased mental distress and lack of face-to-face service administration. Lockdowns undertaken in the matter of public health involved unconsidered trade-offs [11]. Therefore, protecting people who are actually at risk, reopening schools, and rebuilding with a healthy economy are all priorities [11]. 

In the pre-pandemic period, a large-scale, global investigation of sleep disorders in eight countries in Asia and Africa found that roughly 17% of the people in these developing countries suffer from sleep disorders [12]. This number is not different to the average of 20% of the developed world’s populations that are estimated to suffer from some sort of sleep disorder [13]. Due to increased stressors and psychosocial elements, directly and indirectly, resulting from the pandemic, increased stress and anxiety are defined and prominent [14]. Behavioural issues resulting from increased mental discomfort are consequently observed. Insomnia is a possible result of increased anxiety in the ongoing pandemic setting. Anxiety and stress stemming from physical isolation, removal from normative environments, potential infection and other pandemic-related effects could elicit a behavioural response, such as insomnia. As further expressed by Kokou-Kpolou et al. (2020) [15], the prevalence of poor sleep quality and insomnia is highly evident in the pandemic. While the definiteness of the prevalence is yet to be analysed, logical observations and current facts surrounding the mental stress experienced by people during the COVID-19 pandemic support the actuality of the situation. 

According to Simpson and Manber (2020), the quality of sleep decreases, while the regularity of insomnia cases increases under the ongoing global pandemic [14]. Intensified psychological distress relating to isolation, lack of adaptation, medical worries and many more worsen sleep quality and allow for the development of insomnia. As added by Kokou-Kpolou et al. (2020), pre-existing mental issues and consistent stress brought by the pandemic are factors that may greatly affect sleep quality and the development of insomnia [15]. Also, using the protective devices could lead to respiratory and ocular complaints, impairment in job performance, concentration difficulties, and sleep disorders [16]. Other factors, such as age, gender, economic status, educational attainment, location and many more essential roles of varying degrees in the expected quality of sleep and potential outcomes. Additionally, lack of sleep quality may correlate with increased anxiety, fatigue, reliance on destructive coping and stress [14]. 

Psychological mechanisms and deterioration lead to more stress, which creates a cyclic process of continued mental degradation. Quality of sleep, level of anxiety, and other psychological factors need further observations for better understanding and coping, especially considering the massive toll of the pandemic on people’s behaviour and psychological aspects. This study addresses the gap in many essential factors during the pandemic among the general public in the US with the aim of assisting health care providers to assess: (1) the prevalence of poor sleep quality and insomnia, (2) exploring the relationship between sleep quality and other outcomes, including anxiety, fatigue, stress and active coping and (3) finding the predictors of sleep quality. 

## 2. Materials and Methods

This was a cross-sectional study using a publicly available database taken from the Boston College COVID-19 Sleep and Well-Being Dataset https://osf.io/gpxwa/ (accessed on 10 September 2021). The full details of the methodology used are presented here [17]. The Boston College COVID-19 Sleep and Well-Being Dataset includes five different rounds, in which round 1 is launched between March and May 2020. In our analysis, we selected the most recent round that included data about sleep quality and other measures, including insomnia, anxiety, stress, fatigue and coping, which was round 5, which was collected between 22 February–8 March 2021. Inclusion criteria were all English-speaking adults more than 18 years old. 

In this round of assessments, the following previously validated measures were included: Pittsburgh Sleep Quality Index (PSQI) (A 7-item and its scores range from 0 to 21 and a score >5 be considered as a significant sleep disturbance) [18], Generalised Anxiety Disorder (GAD-7) Scale (A 7-item and its scores range from 0 to 21 and a score of 10 or greater indicates anxiety) [19], Insomnia Severity Index (ISI) (A 7-item and its scores range from 0 to 28 and a score >14 be considered as a clinical insomnia) [20], Perceived Stress Scale (A 10-item and its scores range from 0 to 40 and a score of >13 indicates higher perceived stress) [21], John Henryism Active Coping Scale (JHACS) (a 12-item and its scores range from 12 to 60 with higher score indicating a greater coping) [22], PROMIS (Patient-Reported Outcomes Measurement Information System) Fatigue Scale (a 7-item and higher scores indicating greater fatigue) [23]. 

In addition to the collected surveys, we collected demographics and other information concerning level of education, income, Multifactorial Memory Questionnaire (MMQ) Satisfaction, and COVID Impact. All files of the questionnaires and README files in DOCX and PDF format, which include variable descriptions and explanations of all data processing done in cleaned versions of the data sets, are presented in the online database https://osf.io/gpxwa/ (accessed on 10 September 2021). We adhered to the Strengthening the Reporting of Observational studies in Epidemiology (STROPE) guidelines. The primary outcomes were: the prevalence of poor sleep quality and insomnia and the relationship between sleep quality and other outcomes, including anxiety, fatigue, stress and active coping. The secondary outcome was finding the predictors of poor sleep quality. 

### 2.1. Statistical Analysis

Histograms were used to check for outliers in the data, and the Kolmogorov–Smirnov test was used to determine normality. Data were reported as mean and standard deviation (SD) if normally distributed (parametric) and as median and inter-quartile range (IQR) if not normally distributed (non-parametric). We used Spearman’s rank correlation coefficient test for non-parametric variables and Pearson’s correlation coefficient for normally distributed data to analyse the relationships between variables. To identify the characteristics related to poor sleep quality, a univariate analysis was completed. Following the univeritate analysis, we used a multivariate regression analysis adjusted for age and gender to identify the independent factors (JHACS, ISI, PROMIS fatigue scale, PSS, GAD-7, MMQ satisfaction, and COVID-19 positive impact) related to poor sleep quality, with the dependent variable being the PSQI.

### 2.2. Power Calculation

We did not perform a pre-analysis of power since the data set was already there, but based on our regression analysis, our sample size (476) was large enough to get appropriate power for the three major regression analysis models. A minimum of 10 events per variable in a multivariable method of analysis is recommended; thus, a minimum of 100 subjects is recommended to be able to examine 5 to 10 variables in the multivariate regression model.

## 3. Results

A total of 476 subjects were included in the analysis. The mean (SD) age of the study population was 38.8 (17.8) years, and there were more females (85%) than males (Table 1). The population had a mean (SD) score of 6 (3.2) on the PSQI, with 65% having the prevalence of poor sleep quality (defined as a PSQ ≥ 5; *n* = 311). No statistical difference was observed between males and females (*p* = 0.209). 

The mean (SD) score for insomnia was 6.9 (5.2) in the study population, with 55 subjects (11.5%) having clinical insomnia (defined as ISI ≥ 15); of whom 9% had clinical severe insomnia. The prevalence of clinical insomnia did not differ between males and females (*p* = 0.822). 

There were no statistical differences among the other parameters between males and females, except for the PROMIS fatigue scale, as it was greater in females than in males (15.7 [5.3] vs. 17.7 [5.4]; *p* = 0.003). 

### 3.1. Correlations between PSQI and Other Parameters

**Figure 1:**
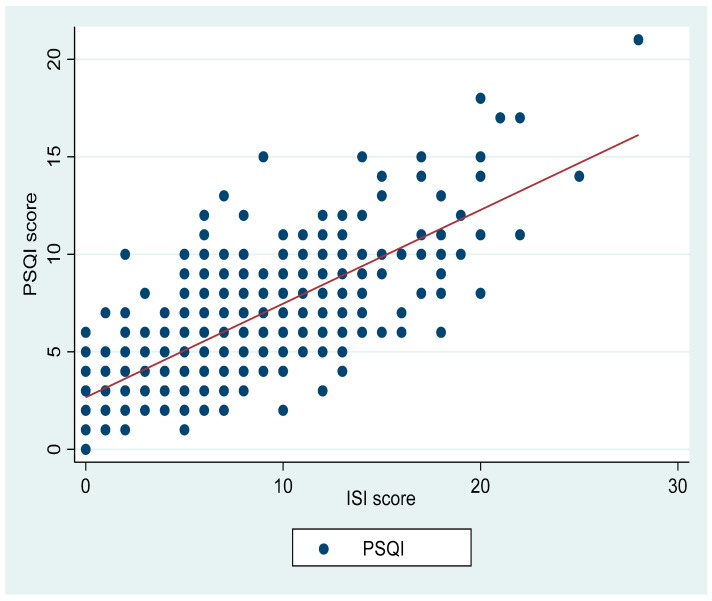
Shows the correlation between PSQI and ISI (r = 0.76, *p* < 0.001).

**Figure 2:**
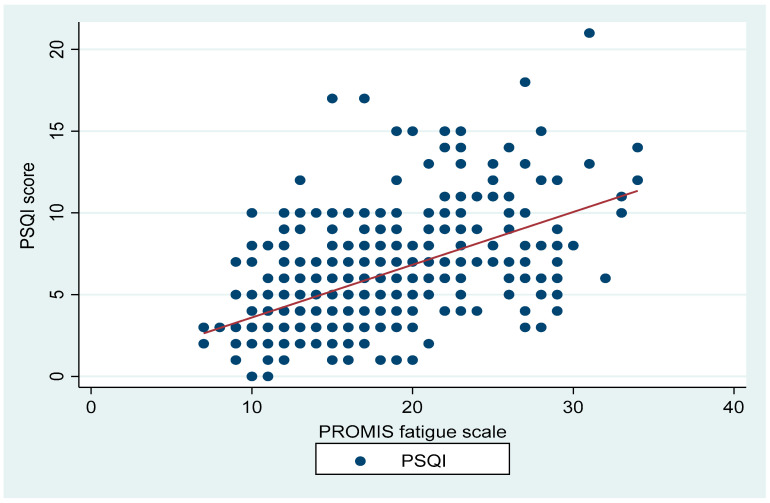
Shows the correlation between the PSQI and PROMIS fatigue scale (r = 0.53, *p* < 0.001).

**Figure 3:**
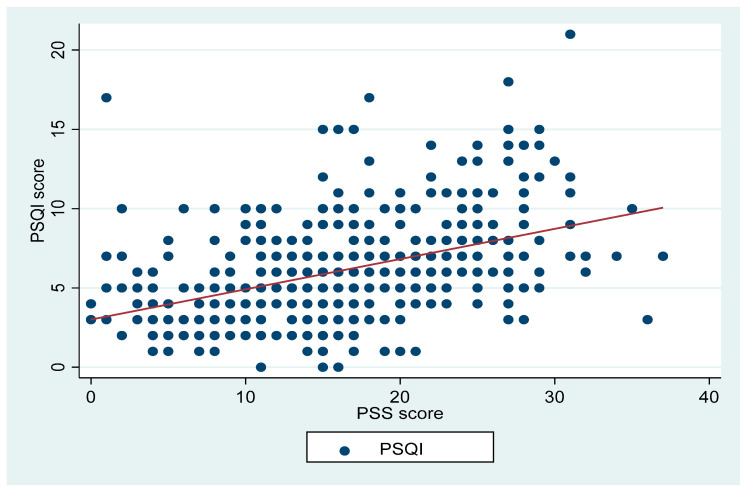
Shows the correlation between the PSQI and PSS (r = 0.44, *p* < 0.001).

There were positive correlations between the PSQI and ISI (r = 0.76, *p* < 0.001; Figure 1), PROMIS fatigue scale (r = 0.53, *p* < 0.001; Figure 2), and PSS (r = 0.44, *p* < 0.001; Figure 3). PSQI was inversely related to the JHAC scale, MMQS and COVID impact, as shown in Table 2.

### 3.2. Associations of PSQI with Other Parameters

There were statistically significant associations between PSQI and the independent variables in the univariate regression models (Table 3). A unit change (increase) in PSQI score was associated with an increase in ISI, PROMIS fatigue scale, PSS and GAD-7 by 0.48, 0.32, 0.19, and 0.32, respectively and a decrease in JHACS, MMQ satisfaction and COVID-19 positive impact by 0.08, 0.06, and 0.12, respectively. These associations remained significant even after adjusting for age and gender. 

In the multivariate regression model, JHACS, ISI, fatigue, PSS and GAD were significant predictors for PSQI, and these variables accounted for 62% of the variance in PSQI, as shown in Table 3.

## 4. Discussion

This is the first study to assess quality of sleep and its association with insomnia, anxiety, fatigue, stress, memory and active coping during the COVID-19 Pandemic. The results of this study highlight the high prevalence of poor sleep quality among both males and females in the study cohort, where 65% of subjects reported poor sleep quality and around 12% of the subjects had clinical insomnia during the pandemic. There were statistically significant associations between PSQI and other measures, including the ISI, GAD-7, PSS, PROMIS Fatigue Scale, JHACS, MMQ satisfaction and COVID Positive, adjusted for age and gender. 

Sleep quality is directly and indirectly impacted by a multitude of cultural, social, psychological, pathophysiological and environmental factors. Poor sleep is linked with negative consequences for physical and mental well-being [24,25,26,27,28], which may have been further exacerbated by the COVID-19 pandemic [29]. In our sample, when the PSQI score increased by one unit, the ISI, PROMIS fatigue scale, PSS and GAD-7 also increased by 0.48, 0.32, 0.19, and 0.32 units, respectively, while there was a decrease in JHACS, MMQ satisfaction and COVID positive impact by 0.08, 0.06, and 0.12 units, respectively. Such findings were expected as those who had poor sleep quality would have more insomnia, fatigue, stress and anxiety. There is a clear link between sleep quality and psychopathology. According to an RCT, greater sleep quality was linked to improved mental health, shedding further light on the relationship between sleep and one’s mental well-being [30]. 

Only 11.5% of the respondents had clinical insomnia, defined as an ISI score of 15 or more. The yields in this exploration were lower than the pre-pandemic prevalence rate in working people, which was estimated at 23% [31]. Notably, the findings were also two and three times lower than those reported in Italian and Greek population studies during the pandemic [32]. This inconsistency may be explained by the variations in the insomnia assessment instruments used, the sociodemographics of the populations, and the timing of data collection, which may also partly contribute to the inconsistency of findings across the studies.

Prior evidence showed that insomnia is significantly higher in females [33] who might be more vulnerable to the impact of socioeconomic factors, including lower income and educational levels [34]. Females are also predisposed to experiencing certain health-related issues such as anxiety, depression and coping strategies [35,36]. Contrary to expectations, this study did not find a significant difference between females and males in insomnia rate or all the other parameters in the analysis except for the PROMIS fatigue scale, which was higher in females by 2 points—an increment that is statistically significant but not necessarily clinically important [37]. Although the reason for the lack of significant gender findings is not entirely clear, this may be partly explained by the study population included in the analysis, which comprised highly educated individuals, with female respondents being the majority of the sample (85%). This imbalance in the distribution between genders may have contributed to reducing the effect of gender in the analyses. Another possible explanation for this is that other confounding factors, including age, level of education and socio-economic status, may have nullified the gender effect in this study. Further sub-analyses to assess different strata of society could help in further understanding the sociodemographic and psychological correlates of insomnia, poor sleep quality, and other parameters. 

Among the various parameters assessed in the current study, ISI had a strong positive correlation with poor sleep quality as assessed by PSQI, while PROMIS and PSS had moderately positive correlations with PSQI. Regarding the associations between poor sleep quality and the other parameters, the PSQI was significantly associated with all the parameters in the analysis model and remained significant even after adjusting for age and gender. While a note of caution is due when interpreting these findings, these observations extend prior research by demonstrating that, during the pandemic, perceived poor sleep quality is positively correlated with self-reported health and psychological symptoms. Such findings align with previous studies showing strong associations between PSQI and ISI, fatigue and psychological symptoms in different populations [38,39]. In the current study, stress and anxiety were associated with poor sleep quality. This finding broadly supports the well-documented work of other studies in this area linking stress and anxiety with sleep quality. One study conducted among college nursing students found that perceived stress mediated the association between sleep quality and symptoms of anxiety [40]. It was also confirmed in another research, which revealed that stress and the pressure to maintain grade point averages may be affecting students’ sleep quality [41].

Boluarte et al. [42] reported that more than 65% of the participants had at least one mental health condition (depression, anxiety or stress symptoms) during the COVID-19 period. In another recent study, Tsang et al. found that participants with higher levels of stress and anxiety were more likely to report shorter sleep durations and poorer sleep quality during the COVID-19 pandemic [43]. 

According to a prospective study, there was a bidirectional association between generalised anxiety and depression, as well as poor sleep hygiene, among the general population [44]. This indicates that such measures can be intertwined over time. Coping is an important strategy for managing prolonged exposure to social stressors, such as COVID-19 [45]. We found that those with fewer coping mechanisms during the pandemic had poor sleep quality. Thus, improving the coping process for individuals would not only have a positive impact on their sleep quality, but also their mental health and well-being. 

When the level of memory satisfaction increased, the sleep quality improved and this could be explained by a neurobiological model suggesting that sleep directly affects the activity of the medial temporal lobes and hippocampus, which then affects the formation of memories [46]. Another possible proxy explanation could be partly related to the respondents’ perceptions of their cognitive performance and sleep quality. In a previous study evaluating cognitive impairment, individuals with insomnia were more dissatisfied with their memory and reported more cognitive difficulties compared to controls [47]. This was also reported in a correlational study in adults without sleep disorders, which found that lower MMQ ability, not satisfaction, scores were linked with increased somnolence during the day and self-reported poor sleep quality [48].

Notwithstanding the insights that the current study highlighted, some limitations call for caution when interpreting the findings. Our ability to generalise the findings is limited due to the cross-sectional nature of the data and the socio-demographics of the study sample, which are over-representative in terms of race and gender. 

The study sample is skewed towards respondents who are female, highly educated, and from Massachusetts in the United States of America. Additionally, the measures in the study relied entirely on self-reported data. It is imperative that future research consider the role of sociodemographic factors and their impact on sleep quality and psychological symptoms. 

## 5. Conclusions

The COVID-19 pandemic has impacted nearly all aspects of everyday life, particularly health and well-being. The current study contributes to the literature by highlighting the high rates of poor sleep quality and its association with insomnia and other psychological symptoms. It also emphasises the need for refocusing policy and public health efforts to address sleep problems that have serious health and economic consequences not only on individuals but also at a public level. As we continue to deconstruct the pandemic’s impact on health and mental well-being, it is critical that we consider investigating pandemic-related health burdens on populations with varying socio-demographic characteristics.

## Figures and Tables

**Table 1:** Demographics and clinical characteristics (*n* = 476).

Variable	Mean (SD Unless Stated Otherwise)
**Age**	38.8 (17.8)
**Gender, *n* (%)**	
Male vs. female	73 (15%) vs. 403 (85%)
**Education, *n* (%)**	
Some high school	2 (0.4%)
High school diploma or GED	15 (3.2%)
Some college	62 (13%)
College degree	125 (26.3%)
Some post-graduate education	50 (10.5%)
Graduate, medical, or professional education	222 (46.6%)
**Income, *n* (%)**	
$0–$25,000	4 (10.3%)
$25,001–$50,000	78 (16.4%)
$50,001–$75,000	85 (17.9%)
$75,001–$100,000	84 (17.7%)
$100,001–$150,000	54 (11.3%)
$150,001–$250,000	54 (11.3%)
$250,000+	39 (8.2%)
**Clinical parameters**	
PSQI	6 (3.2)
JHACS	43.6 (6.3)
ISI	6.9 (5.2)
PROMIS Fatigue Scale	17.4 (5.4)
PSS	15.8 (7.6)
GAD-7	5.3 (4.8)
MMQ Satisfaction	48.4 (14.6)
COVID-19 Positive Impact	20 (5.7)
**Poor sleep quality, *n* (%)**	311/476 (65%)
**Clinical Insomnia, *n* (%)**	55/476 (11.5%)

Abbreviations: PSQI: Pittsburgh Sleep Quality Index; JHACS: John Henryism Active Coping Scale; ISI: Insomnia Severity Index; PROMIS: Patient-Reported Outcomes Measurement Information System; PSS: Perceived Stress Scale; GAD-7: Generalised Anxiety Disorder-7; MMQ: Multifactorial Memory.

**Table 2:** Correlations between Pittsburgh Sleep Quality Index and other parameters.

Variable		
	Pittsburgh Sleep Quality Index	
	r	*p* Value
**JHACS**	−0.1637	<0.001
**ISI**	0.7691	<0.001
**PROMIS Fatigue Scale**	0.5358	<0.001
**PSS**	0.4456	<0.001
**GAD-7**	0.4660	<0.001
**MMQ Satisfaction**	−0.3152	<0.001
**COVID Positive Impact**	−0.1908	<0.001

Abbreviations: PSQI: Pittsburgh Sleep Quality Index; JHACS; John Henryism Active Coping Scale; ISI: Insomnia Severity Index; PRMIS: Patient-Reported Outcomes Measurement Information System; PSS: Perceived Stress Scale; GAD-7: Generalised Anxiety Disorder-7; MMQ: Multifactorial Memory.

**Table 3:** Results of the univariate and multivariate regression analyses.

Dependent Variable: PSQI						
	Model 1: β (95% CI)	*p* Value	Model 2: β (95% CI)	*p* Value	Model 3: β (95% CI)	*p* Value
**JHACS**	−0.08 (−0.13 to −0.03)	0.001	−0.08 (−0.13 to −0.03)	0.001	−0.03 −0.07 to 0.004	0.005
**ISI**	0.48 (0.44 to 0.51)	<0.001	0.48 (0.44 to 0.52)	<0.001	0.4 0.35 to 0.44	<0.001
**PROMIS fatigue scale**	0.32 (0.27 to 037)	<0.001	0.33 (0.28 to 0.38)	<0.001	0.05 0.005 to 0.1	0.03
**PSS**	0.19 (0.15 to 0.22)	<0.001	0.20 (0.17 to 0.24)	<0.001	0.004 −0.037 to 0.05	0.83
**GAD-7**	0.32 (0.26 to 0.37)	<0.001	0.32 (0.27 to 0.38)	<0.001	0.09 0.02 to 0.01	0.013
**MMQ**	−0.06 (−0.08 to −0.05)	<0.001	−0.06 (−0.08 to 0.−05)	<0.001	−0.002 −0.02 to 0.01	0.52
**COVID-19 impact**	−0.12 (−0.16 to −0.05)	<0.001	−0.10 (−0.16 to −0.05)	<0.001	−0.01 −0.04 to 0.02	0.86

**Model 1**: Univariate association of PSQI and other variables. **Model 2**: Associations of PSQI and other variables adjusted for age and gender. **Model 3**: Multivariable associations of PSQ as the dependent variable and JHACS, ISI, PROMIS fatigue scale, PSS, GAD-7, MMQ satisfaction, and COVID-19 positive impact as independent variables. **Abbreviations:** CI: confidence interval; PSQI: Pittsburgh Sleep Quality Index; JHACS; John Henryism Active Coping Scale; ISI: Insomnia Severity Index; PRMIS: Patient-Reported Outcomes Measurement Information System; PSS: Perceived Stress Scale; GAD: Generalised Anxiety Disorder-7; MMQ: Multifactorial Memory.

## Data Availability

It can be accessed via a publicly available database taken from the Boston College COVID-19 Sleep and Well-Being Dataset https://osf.io/gpxwa/ (accessed on 10 September 2021).
